# Trends of Female Breast Cancer Incidence, Mortality, and Survival in Fujian Province of China: 2011–2020 and Projection to 2025

**DOI:** 10.1002/cam4.71033

**Published:** 2025-07-11

**Authors:** Yeying Wen, Jingyu Ma, Zhisheng Xiang, Yongtian Lin, Yongying Huang, Linying Liu, Yan Zhou, Yang Sun

**Affiliations:** ^1^ Department of Epidemiology Clinical Oncology School of Fujian Medical University, Fujian Cancer Hospital Fuzhou Fujian China; ^2^ Department of Gynecology Clinical Oncology School of Fujian Medical University, Fujian Cancer Hospital Fuzhou Fujian China

**Keywords:** breast cancer, population‐based study, prediction, temporal trends

## Abstract

**Background:**

Breast cancer was the second most common cancer and the fifth leading cause of cancer deaths among women in China, with increasing trends. Evaluating breast cancer trends and predicting future burdens can inform prevention strategies. This study aimed to analyze the trends in female breast cancer incidence, mortality, and survival in Fujian Province, southeastern China, between 2011 and 2020, and to project the future burden through 2025.

**Methods:**

Population‐based cancer registry data from Fujian Province were collected during 2011–2020, with survival follow‐up extending through March 2022, covering approximately 2.59 million women. Age‐standardized incidence rates (ASIR) and mortality rates (ASMR) were calculated using Segi's world standard population. Temporal trends were assessed using Joinpoint regression analysis to determine average annual percentage change (AAPC). Relative survival were computed as the ratio of observed survival to expected survival. Autoregressive Integrated Moving Average (ARIMA) model was employed to predict incidence and mortality for 2021–2025.

**Results:**

During 2011–2020, 8047 female breast cancer cases and 1754 deaths were recorded. The ASIR increased from 19.34/100,000 to 38.73/100,000, with an AAPC of 7.4% (95% CI: 3.6%–11.4%). The ASMR increased from 3.37/100,000 to 6.19/100,000, with an AAPC of 8.3% (95% CI: 4.3%–12.4%). Rural areas showed more rapid increases in both incidence and mortality than in urban areas. In 2011–2014, 2015–2017, and 2018–2020, the age‐standardized 5‐year relative survival rates were 64.10% (95% CI: 59.85–68.02), 69.35% (95% CI: 64.62–73.57), and 72.94% (95% CI: 68.5–76.86), with higher survival in urban areas and younger age groups. The ARIMA models projected continued increases in both ASIR and ASMR through 2025, reaching 46.41/100,000 and 8.46/100,000, respectively.

**Conclusion:**

Breast cancer constitutes an escalating public health challenge in Fujian Province with disparities across age groups and urban and rural areas. Future strategies should prioritize healthcare equity and regional resource allocation to reduce mortality and enhance survival outcomes.

## 1 Introduction

Breast cancer is the most frequently diagnosed cancer and the leading cause of cancer deaths among women worldwide. According to GLOBOCAN 2022 estimates [1], breast cancer accounted for 2.2 million new cases and 66.6 thousand deaths in 2022. This indicates that breast cancer is the most prevalent cancer and the leading cause of mortality among women globally. Compared with other countries, the age‐standardized incidence and mortality rates of female breast cancer in China were relatively low [2]. Nevertheless, the rising trend of breast cancer in Chinese women is more alarming than the current absolute burden indicators: The incidence of breast cancer increased by 3.3% per year and the mortality increased by 1.0% per year between 2000 and 2015, and is projected to increase by more than 11% by 2030 [3]. Over the past decades, the incidence and mortality of female breast cancer in China have been steadily rising, particularly in rural areas [4]. With rapid economic development, increasing aging, population growth, and the growing prevalence of major risk factors, the burden of breast cancer is expected to continue increasing in the coming years [5]. Assessing trends in female breast cancer and projecting its future burden will provide valuable insights into cancer prevention and control statistics.

China is a vast country, and the prevalence of female breast cancer varies widely across geographic regions [6]. For this report, we focus on the trends of the incidence, mortality, and survival of female breast cancer in Fujian Province, southeastern China. Fujian, situated on the southeastern coast of China, has a population of 41.8 million and a per capita gross domestic product (GDP) of ¥129,865 (about $18,089) in 2023 [7]. Breast cancer has emerged as the second most prevalent cancer among women across the region [8], with rising incidence and mortality rates constituting a major public health burden.

Therefore, to understand the epidemiology of female breast cancer in Fujian Province, this study analyzed the trends of incidence, mortality, and survival of female breast cancer from 2011 to 2020, and projected the future burden through 2025. These findings provide a scientific basis for health policy reform and optimization of healthcare resources allocation.

## 2 Materials and Methods

### 2.1 Data Sources

The Fujian Cancer Prevention and Control Office is responsible for cancer surveillance in Fujian Province and regularly collects statistics from each population‐based cancer registry. This study utilized data from seven registries of the Fujian Cancer Prevention and Control Office, covering about 2.59 million women (13.4% of the province's female population) in 2020.

This research enrolled in the incidence and mortality of patients diagnosed with female breast cancer from January 1, 2011, to December 31, 2020. All cases were followed up until March 31, 2022, for survival status. The patient survival status was assessed by both active and passive follow‐up methods. The passive follow‐up is matched cause‐of‐death surveillance data and cancer incidence data performed by registry staff to complement time and survival outcome information. For cases that can not correspond to cause‐of‐death surveillance data, active follow‐up methods were employed to collect survival data. Active follow‐up consists of phone or home visits to confirm case survival. Demographic statistics were obtained from the Public Security Household Registration Department. The 3rd edition of the International Classification of Diseases for Oncology (ICD‐O‐3) and the10th edition of the International Classification of Diseases (ICD‐10) were used for the coding of breast cancer. In this research, breast cancer was identified by ICD‐10 codes of C50.

### 2.2 Quality Control and Exclusions

According to the Guidebook for Cancer Registration in China (2016) [9], only cases with the right logistic relationships will be validated and reported. We used the International Agency for Research on Cancer/International Association of Cancer Registries (IARC/IACR) [10, 11] quality standards to assess cancer data metrics. Detailed information has been published in a previous study [12]. The completeness, comparability, and validity of the data were evaluated according to indicators such as the percentage of cases morphologically verified (MV%), the percentage of death certificate‐only cases (DCO%), and the mortality to incidence (M/I) ratio. In this research, the MV% of female breast cancer was 87.13%, the DCO% was 0.10%, and the M/I was 0.20, as provided in the appendix (Table S1). Cases were eliminated from the analysis if they were based solely on death certificates or if there were more than two primary malignancies in a single case.

### 2.3 Statistical Analysis

Data from seven cancer registries that met quality control criteria were pooled and analyzed. The number of new cases or deaths is divided by the population in the same registration area to compute the crude incidence and mortality rates. Age‐standardized incidence rates (ASIR) and age‐standardized mortality rates (ASMR) were calculated using Segi's world standard population [13]. SAS 9.0 was utilized to calculate incidence and mortality rates. Joinpoint regression models were applied to generate estimates of time trends in incidence and mortality, calculating the average annual percentage change (AAPC) and 95% confidence interval (CI) [14]. To evaluate changes in differences between urban and rural areas, we divided the registries into urban and rural areas and compared differences between urban and rural areas based on the regional classification of the National Bureau of Statistics of China.

We used the relative survival as the primary survival indicator, which was calculated as the ratio of the observed survival rate to the expected survival rate from a comparable group of the general population. We estimated expected survival based on the Ederer II [15] method. Abridged life tables were smoothed to complete life tables and extended to the age of 99 years using the Elandt–Johnson method [16]. The diagnostic years were categorized into three calendar periods: 2011–2014, 2015–2017, and 2018–2020. For the period 2011–2014, relative survival was computed using the cohort method; for 2015–2017, the complete method was applied; and for 2018–2020, the period method was utilized to predict relative survival [17]. The age‐standardized relative survival rates of breast cancer were calculated based on the International Cancer Survival Standards 1 (ICSS1): 0–44 years, 7%; 45–54 years, 12%; 55–64 years, 23%; 65–74 years, 29%; and 75–99 years, 29% [18]. We also characterized the temporal trend in incidence, mortality, and survival in these five major age cohorts. Relative survival was calculated using the *Strs* package via Stata 12.0.

The autoregressive integrated moving average (ARIMA) model was employed to forecast the trend of breast cancer incidence and mortality in Fujian Province for 2021–2025. The ARIMA model is a widely used method of time series analysis that integrates autoregression (AR), integration (I), and moving average (MA) components. These components effectively catch trending and periodic patterns in time series data and provide reliable forecasts of future trends on the basis of current data [19]. To assess the predictive accuracy of the model, we used established indicators such as root mean square error (RMSE) and mean absolute percentage error (MAPE).

## 3 Results

### 3.1 Overall Female Breast Cancer Incidence and Mortality

There were 8047 patients diagnosed with breast cancer from 2011 to 2020, with an average age of 60.74 years. Among them, 69.4% were from urban areas and 30.6% were from rural areas (Table S2). The crude incidence and ASIR of breast cancer increased from 23.50/100,000 and 19.34/100,000 in 2011 to 53.89/100,000 and 38.73/100,000 in 2020, respectively (Table 1). For mortality, 1754 patients died of breast cancer during 2011 to 2020, with an average age of 64.83 years. Among them, 65.7% were from urban areas and 34.3% were from rural areas. The crude mortality and ASMR increased from 4.09/100,000 and 3.37/100,000 in 2011 to 9.12/100,000 and 6.19/100,000 in 2020. For urban and rural areas, the breast cancer incidence and mortality rates were higher in urban areas than in rural areas during the same years (Table 2).

**TABLE 1:** The incidence of female breast cancer in Fujian Province, 2011–2020.

Year	Total			Urban			Rural		
	Case	Crude rate (1/10^5^)	ASIR (1/10^5^)	Case	Crude rate (1/10^5^)	ASIR (1/10^5^)	Case	Crude rate (1/10^5^)	ASIR (1/10^5^)
2011	489	23.50	19.34	349	30.52	23.50	140	14.94	13.55
2012	529	24.99	19.34	363	30.90	23.04	166	17.62	14.33
2013	522	23.92	18.18	363	29.52	21.63	159	16.70	13.38
2014	592	26.74	20.66	432	34.61	26.08	160	16.56	13.18
2015	672	29.55	22.11	490	37.70	28.13	182	18.68	14.06
2016	801	34.62	25.17	579	43.62	31.30	222	22.51	16.65
2017	789	33.45	24.61	516	37.81	28.59	273	27.46	19.60
2018	1068	43.39	30.83	719	49.22	35.17	349	34.87	24.75
2019	1185	47.27	33.67	798	53.14	38.29	387	38.49	26.99
2020	1400	53.89	38.73	979	61.51	45.11	421	41.85	29.21

Abbreviation: ASIR, age‐standardized incidence rates.

**TABLE 2:** The mortality of female breast cancer in Fujian Province, 2011–2020.

Year	Total			Urban			Rural		
	Deaths	Crude rate (1/10^5^)	ASMR (1/10^5^)	Deaths	Crude rate (1/10^5^)	ASMR (1/10^5^)	Deaths	Crude rate (1/10^5^)	ASMR (1/10^5^)
2011	85	4.09	3.37	58	5.07	4.00	27	2.88	2.50
2012	100	4.72	3.73	68	5.79	4.51	32	3.40	2.66
2013	129	5.91	4.31	81	6.59	4.59	48	5.04	3.96
2014	163	7.36	5.72	104	8.33	6.26	59	6.11	5.05
2015	173	7.61	5.40	120	9.23	6.52	53	5.44	3.94
2016	185	8.00	5.53	132	9.94	6.68	53	5.37	3.97
2017	214	9.07	6.62	147	10.77	8.08	67	6.74	4.78
2018	232	9.42	6.57	150	10.27	7.19	82	8.19	5.75
2019	236	9.41	6.42	147	9.79	6.67	89	8.85	6.09
2020	237	9.12	6.19	146	9.17	6.30	91	9.05	6.05

Abbreviation: ASMR, age‐standardized mortality rate.

The age‐specific incidence rate was relatively low before age 44 years, and gradually increased after age 45 years, peaked at the age group of 45–54 years, and then slowly decreased. The age‐specific mortality rate was relatively low before age 44 years, while it increased after age 45 years, and reached the peak in the 75+ age group (Figure S1).

### 3.2 Trends in Female Breast Cancer Incidence and Mortality

The age‐standardized rates (ASR) of female breast cancer incidence in seven registries in Fujian Province showed an upward trend from 2011 to 2020, with an increase of 7.4% per year (95% CI: 3.6% to 11.4%). The age‐specific incidence rates of female breast cancer increased significantly in all age groups except the > 75 years group, with the greatest increase in the 0–44 year age group (AAPC: 10.5%, 95% CI: 8.2% to 12.8%). By urban and rural areas, the incidence rate of female breast cancer increased by 7.7% in urban areas (95% CI: 5.4% to 10.0%) and 9.0% in rural areas (95% CI: 5.9% to 12.2%). We also analyzed long‐term trends in female breast cancer mortality over the period 2001–2020. During 2011 to 2020, the ASRs of female breast cancer mortality increased by 8.3% per year (95% CI: 4.3% to 12.4%). Analysis of age‐specific mortality rates showed a significant increase in all age groups, with the largest increase in the age group beyond 75 years (AAPC: 18.2%, 95% CI: 7.2% to 30.3%). The increasing trends were clearer in rural areas, with AAPC of 5.0% (95% CI: 1.0% to 9.2%) in urban areas, and AAPC of 9.8% (95% CI: 5.7 to 14.1%) in rural areas, respectively (Table 3).

**TABLE 3:** Trend analysis for incidence and mortality of female breast cancer overall, by age group and area, 2011–2020.

Group	Incidence		mortality	
	AAPC (%)	95% CI	AAPC (%)	95% CI
Total	7.4^a^	3.6–11.4	8.3^a^	4.3–12.4
Age group				
0–44	10.5^a^	8.2–12.8	6.7^a^	1.6–12.1
45–54	9.6^a^	6.7–12.5	7.4^a^	0.9–14.4
55–64	7.8^a^	4.7–10.9	7.5^a^	3.0–12.2
65–74	6.7^a^	2.4–11.1	8.9^a^	2.5–15.6
≥ 75	4.2	−2.7‐11.5	18.2^a^	7.2–30.3
Area of residence				
Urban	7.7^a^	5.4–10.0	5.0^a^	1.0–9.2
Rural	9.0^a^	5.9–12.2	9.8^a^	5.7–14.1

^a^ The AAPC is significantly different from zero (*p* < 0.05).

Abbreviaitons: AAPC, average annual percentage change; CI, confidence interval.

### 3.3 Survival of Breast Cancer

Overall, the age‐standardized 5‐year relative survival rates for breast cancer were 64.10% (95% CI: 59.85 to 68.02), 69.35% (95% CI: 64.62 to 73.57), and 72.94% (95% CI: 68.5 to 76.86) in 2011–2014, 2015–2017, and 2018–2020, respectively. In all three time periods, the age‐standardized 5‐year relative survival rate of cancer patients was higher in urban areas than that in rural areas (Table 4).

**TABLE 4:** Overall 5‐year relative survival rates of female breast cancer.

	2011–2014		2015–2017		2018–2020	
	Crude RS (95% CI)	Adjusted RS (95% CI)	Crude RS (95% CI)	Adjusted RS (95% CI)	Crude RS (95% CI)	Adjusted RS (95% CI)
All	70.65 (68.49–72.7)	64.10 (59.85–68.02)	78.63 (76.69–80.45)	69.35 (64.62–73.57)	79.29 (77.42–81.05)	72.94 (68.5–76.86)
Urban	73.95 (71.44–76.30)	67.06 (62.20–71.44)	80.80 (78.54–82.87)	69.50 (63.91–74.4)	82.45 (80.32–84.4)	75.88 (70.56–80.37)
Rural	62.61 (58.41–66.54)	55.35 (46.35–63.44)	73.43 (69.55–76.94)	68.60 (59.34–76.17)	71.53 (67.57–75.13)	65.05 (56.37–72.43)

Abbreviations: CI, confidence interval; RS, relative survival.

**FIGURE 1:**
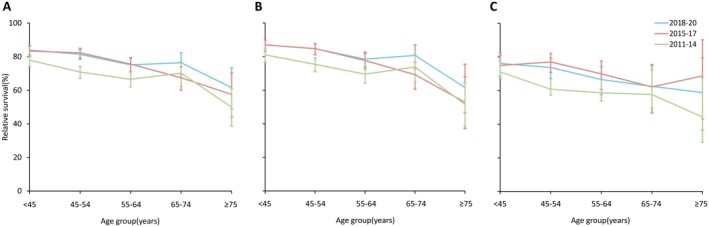
5‐year relative survival of female breast cancer by age. (A) Total cases; (B) urban cases; (C) rural cases.

For different age groups, the 5‐year relative survival rate was generally lower for older patients than for younger patients over the three time periods. In 2018–2020, the 5‐year relative survival rate for patients below the age of 45 years was 83.97%, whereas the rate for patients 75 years of age and older was 61.57%. There was an absolute difference of 22.40% between the two groups. The 5‐year relative survival rate increased in all age groups between 2011 and 2020. When stratified by urban and rural areas, survival growth was greater in urban areas than in rural areas (Figure 1).

### 3.4 Prediction of Breast Cancer Incidence and Mortality

**FIGURE 2:**
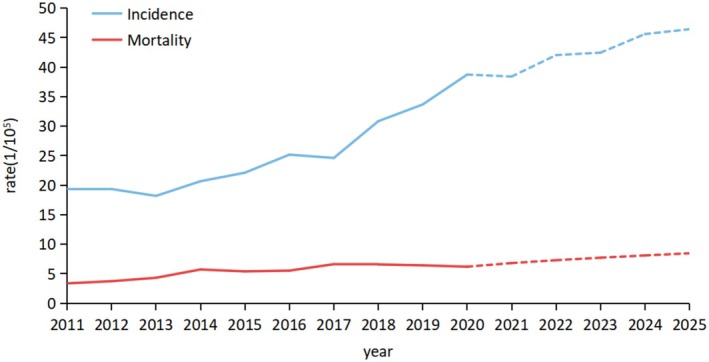
The age‐standardized incidence and mortality of female breast cancer predicted by ARIMA model, 2021–2025.

The ARIMA models of prospective trends in breast cancer incidence and mortality up to 2025 were presented in Figure 2 and Table S3. As can be seen, the expected incidence and mortality rates were projected to continue to increase from 2020 to 2025. The incidence of breast cancer in 2025 was expected to increase to 46.41/100,000 (RMSE: 1.92 MAPE: 5.47), and the mortality in 2025 was expected to increase to 8.46/100,000 (RMSE: 0.56 MAPE: 6.945).

## 4 Discussion

Using the most current cancer registry data from seven cancer registries in Fujian Province, we report comprehensive population‐based information on female breast cancer incidence, mortality, and survival in covered areas of Fujian Province from 2011 to 2020 and project the cancer burden to 2025. The temporal trends of ASIR and ASMR increase consistently during 2011–2020, particularly in rural areas. The age‐standardized 5‐year relative survival rate for female breast cancer was higher in 2018–2020 than in 2011–2014. The disease burden of female breast cancer in Fujian Province is still forecast to increase by 2025, with ASIR and ASMR increasing to 46.41/100,000 and 8.46/100,000, respectively.

The results of this study showed that the ASIR of female breast cancer in Fujian Province showed an increasing trend from 19.34/100,000 in 2011 to 38.73/100,000 in 2020, with an AAPC of 7.4%. The ASIR in Fujian Province was higher than the average level in China [20], but significantly lower than that of developed countries [1]. However, the persistent upward trends and the large population base pose a substantial challenge for effective breast cancer control within the healthcare system. Over the past few decades, similar to other developed countries, China has experienced rapid economic growth, sociocultural, and demographic transformations. These shifts have also led to alterations in lifestyle patterns. Studies have demonstrated that the rising incidence of female breast cancer in transitional countries is associated with a rise in urbanized lifestyle risk factors, for instance fewer births, older age at first birth, reduced breastfeeding time [21], early age at menarche, older age at menopause [22], insufficient physical activity [23], use of oral contraceptives, and hormone replacement therapy [24].

The prevalence of screening is also one of the major factors influencing the incidence of breast cancer. Compared to East Asian regions with similar lifestyle and culture, the ASIR in Fujian Province is lower than that of Japan and South Korea [2]. Possible explanations for this difference include China's younger population [25] and comparatively low screening coverage [26]. The participation rates in female breast cancer screening in Japan and Korea were 48.8% and 56.5% [27, 28], higher than the 30.9% in China [29]. To address this screening gap and improve early detection rates, the Chinese government has implemented comprehensive national screening initiatives. Since 2009, the former Ministry of Health, the Ministry of Finance and the All‐China Women's Federation have jointly carried out a project project entitled “Rural Women's Two Cancers (Cervical Cancer and Breast Cancer) Screening,” providing free breast cancer screening for rural women aged 35–64 years. This initiative was elevated to a major national public health project and subsequently integrated into a basic public health project in 2019 [30, 31]. The program has now been extended to urban and rural areas, covering more than 2600 counties, districts and cities nationwide. Although these policy interventions may help increase screening rates, further efforts will be needed to achieve the screening rates already attained by neighboring countries.

Breast cancer ranks as the fifth leading cause of cancer‐related deaths among women in Fujian Province in 2020. From 2011 to 2020, the ASMR in Fujian Province increased from 3.37/100,000 to 6.19/100,000, with an AAPC of 8.3%. The combined incidence and mortality situation shows that the mortality to incidence ratio of female breast cancer declined from 0.17 to 0.15 in 2011–2020, reflecting the positive results achieved in the prevention and treatment of breast cancer and paralleling the improvement in the survival rate.

Effective early diagnosis, standardized breast cancer management, and further therapeutic advances have contributed to reducing mortality [32]. Although the number of detected cases of early‐stage breast cancer has increased significantly, the impact of screening on mortality reduction remains complex and debated in the literature. Several studies evaluating population‐based screening programs have yielded mixed results regarding effectiveness in reducing late‐stage disease, with some studies raising concerns about overdiagnosis and questioning the magnitude of mortality reduction [33, 34]. Nevertheless, delayed diagnosis and treatment consistently lead to poor prognosis. Delayed diagnosis and treatment for breast cancer can also lead to a poor prognosis. Studies indicate that 20.1% of breast cancer patients in China are diagnosed at stage III and IV, with higher proportions observed among women from economically underdeveloped areas [35]. Treatment delays and long waiting times before first treatment, particularly when resulting in stage progression or additional therapy complications, can lead to a worse prognostic status. The Surveillance Epidemiology and End Results (SEER) demonstrate that delays in definitive surgery exceeding 60 days are associated with a 45% increase in cancer‐specific mortality [36]. Therefore, it is crucial to build a sound and fair healthcare system and to promote multidisciplinary collaboration, which in turn will reduce breast cancer mortality and enhance the quality of life of surviving patients.

From 2011 to 2020, the age‐standardized 5‐year relative survival rate of female breast cancer in Fujian Province increased from 64.10% to 72.94%. This improvement likely reflects multiple contributing factors. Population‐based survival rates may benefit from higher screening utilization through earlier stage detection, though the relationship between screening and survival outcomes requires careful interpretation considering lead‐time bias and other confounding factors [37]. Beyond screening effects, effective and structured breast cancer management has also had an important place in avoiding recurrence and metastasis as well as in extending survival. In recent years, molecularly targeted therapies, including endocrine inhibitors and human epidermal growth factor receptor 2 (HER2), have changed traditional breast cancer therapy through lower toxicity, improved response rates, and enhanced prognoses [38]. Additionally, with China's national medical reform launched in 2009, basic medical insurance coverage has been extended and the national reimbursement drug list has supported more necessary anticancer drugs, thereby reducing the financial barriers to treatment and contributing to improved survival outcomes [39].

In our age‐specific analyses, younger women (0–44 years) had the greatest trend of incidence throughout the observation period, consistent with recent studies globally that have shown an increase in breast cancer incidence in premenopausal women [40]. The potential causes of the trends seen in young populations are multiple and reflect changing modifiable exposure to risk factors (such as reproductive‐related factors, lifestyle habits) [41].

Although breast cancer incidence and mortality have increased across the province, the rise has been more pronounced in rural areas. One contributing factor is the national free screening program for breast and cervical cancer, which was initially implemented in rural areas and led to increased detection and reported incidence. In addition, recent lifestyle changes [42], including dietary shifts and declining physical activity, may have further contributed to rising incidence. However, rural areas still face challenges such as limited access to diagnostic and treatment services [43], lower health awareness [44], and financial or geographic barriers to care. These findings highlight the need to strengthen rural screening coverage, improve access to timely treatment, and enhance health education tailored to rural populations.

This study presents an updated analysis of female breast cancer incidence, mortality, and survival trends over a 10‐year period and across urban and rural areas using reliable population‐based data provided by the cancer registries in Fujian Province, contributing to the epidemiologic profile of female breast cancer in Fujian Province, southeastern China. Furthermore, we presented a projections of future female breast cancer incidence and mortality rates, which provide valuable reference information for breast cancer prevention and control in the region. However, certain limitations warrant consideration when interpreting these findings. First, the study data were derived from the coverage of the population‐based cancer registries in Fujian Province rather than the entire provincial population. Nonetheless, with the current level of coverage, population‐based cancer registration was recognized as being able to offer a scientific basis for cancer control policies [45]. Second, the lack of data on clinicopathological characteristics, such as anatomical sites and histological subtypes, limited our ability to perform more comprehensive subgroup analyses. Third, our ARIMA projections assume continuation of observed trends and may not account for potential future changes in screening programs, lifestyle factors, or healthcare interventions that could significantly alter breast cancer epidemiology. Therefore, our projections should be interpreted alongside real‐world policy developments. For longer term trend prediction, future work might incorporate machine learning, deep learning, or ensemble approaches to improve robustness and accuracy.

## 5 Conclusions

The burden of female breast cancer in Fujian Province has continued to increase between 2011 and 2020 and is projected to continue rising over the next five years. It is critical to enhance screening coverage, optimize healthcare resource allocation, improve public health awareness, and advance early diagnosis and precision treatment to address the growing burden of breast cancer and improve patients' quality of life.

## Author Contributions

**Yeying Wen:** conceptualization, methodology, writing – original draft. **Jingyu Ma:** data curation, methodology. **Zhisheng Xiang:** data curation, formal analysis. **Yongtian Lin:** formal analysis. **Yongying Huang:** formal analysis. **Linying Liu:** data curation. **Yan Zhou:** project administration, writing – review and editing. **Yang Sun:** conceptualization, project administration, writing – review and editing.

## Ethics Statement

The study was approved by the Institutional Review Board of Fujian Cancer Hospital (No. 2017–047‐01). The need for informed consent was waived by the Research Ethics Review Committee of Fujian Cancer Hospital due to the retrospective and anonymous study design. All methods were performed in accordance with the relevant guidelines and regulations.

## Conflicts of Interest

The authors declare no conflicts of interest.

## Supporting information


Figure S1.

Data S1.

## Data Availability

The data that support the findings of this study are available on request from the corresponding author. The data are not publicly available due to privacy or ethical restrictions.
