# Supplementary figures and images for: Trends of Female Breast Cancer Incidence, Mortality, and Survival in Fujian Province of China: 2011–2020 and Projection to 2025
Source: Cancer Med. 2025 Jul 11;14(13):e71033. doi: 10.1002/cam4.71033 (PMC12246830; doi:10.1002/cam4.71033)

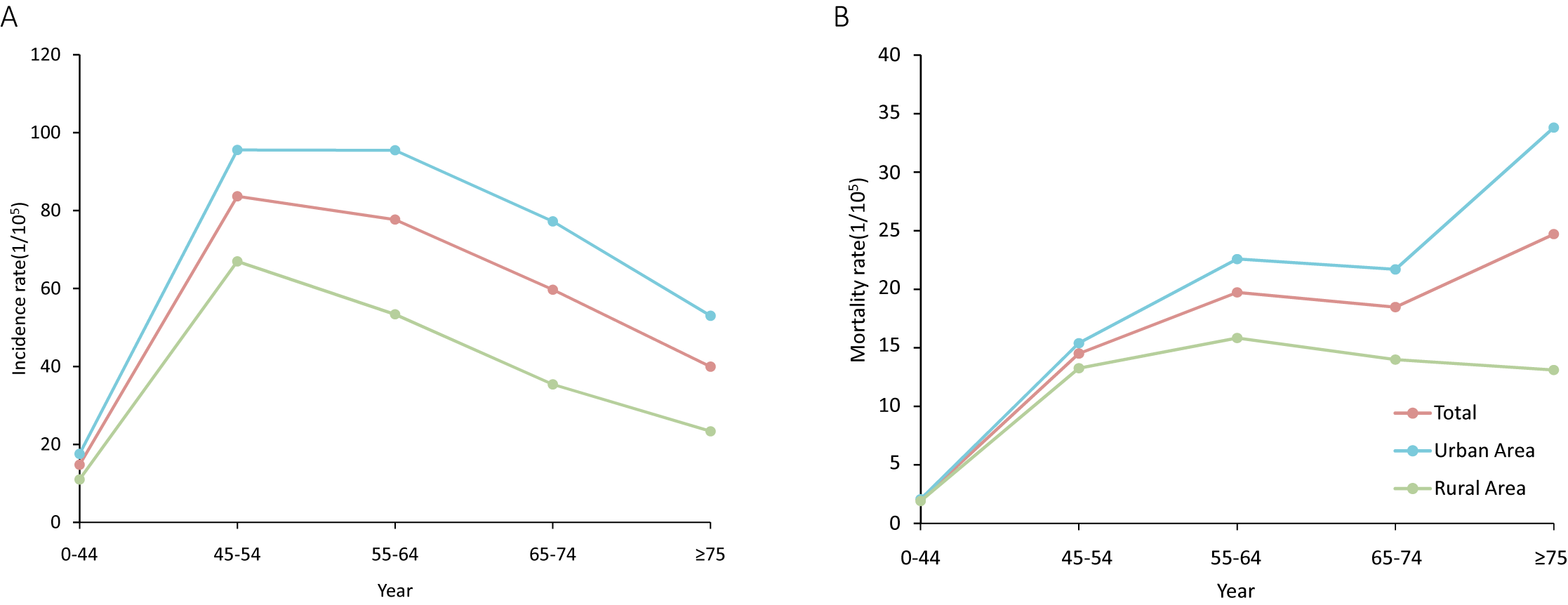

Supplement: Supplementary file 1 — Figure S1. [file CAM4-14-e71033-s002.tif]
