# Supplementary material for: Trends of Female Breast Cancer Incidence, Mortality, and Survival in Fujian Province of China: 2011–2020 and Projection to 2025
Source: Cancer Med. 2025 Jul 11;14(13):e71033. doi: 10.1002/cam4.71033 (PMC12246830; doi:10.1002/cam4.71033)
Supplement: Supplementary file 2 — Data S1. [file CAM4-14-e71033-s001.docx]

**Table S1** Indicators of data quality for the 7 cancer registries in Fujian Province, 2011-2020

| Year | MV% | DCO% | M/I |
| --- | --- | --- | --- |
| 2011 | 86.09 | 0.00 | 0.17 |
| 2012 | 85.82 | 0.38 | 0.18 |
| 2013 | 84.48 | 0.00 | 0.24 |
| 2014 | 84.63 | 0.00 | 0.26 |
| 2015 | 85.42 | 0.15 | 0.24 |
| 2016 | 85.64 | 0.25 | 0.21 |
| 2017 | 88.85 | 0.25 | 0.25 |
| 2018 | 91.39 | 0.00 | 0.20 |
| 2019 | 87.17 | 0.08 | 0.19 |
| 2020 | 87.43 | 0.00 | 0.16 |
| 2011-2020 | 87.13 | 0.10 | 0.20 |

Abbreviations:MV%, the percentage of cases morphologically verified; DCO%, the percentage of death certificate-only cases; M/I, mortality to incidence ratio.

**Table S2** Distribution of registries of the study

| Registry | Area | Cases of incidence | Deaths cases |
| --- | --- | --- | --- |
|  |  |  |  |
| Changle | Rural | 982 | 214 |
| Hanjiang | Urban | 676 | 160 |
| Hui’an | Rural | 934 | 274 |
| Tong’an | Urban | 495 | 90 |
| Xiamen | Urban | 3,908 | 794 |
| Xiang’an | Urban | 509 | 109 |
| Yongding | Rural | 543 | 113 |
| All | Urban and rural | 8,047 | 1,754 |

**Table S3** The age-standardized incidence and mortality of female breast cancer predicted by ARIMA model, 2021-2025

| Year | ASIR（1/10^5^） | ASMR（1/10^5^） |
| --- | --- | --- |
| 2021 | 38.40 | 6.80 |
| 2022 | 42.03 | 7.29 |
| 2023 | 42.44 | 7.71 |
| 2024 | 45.59 | 8.10 |
| 2025 | 46.41 | 8.46 |

Abbreviations: ASIR, age-standardized incidence rates; ASMR, age-standardized mortality rates.

**
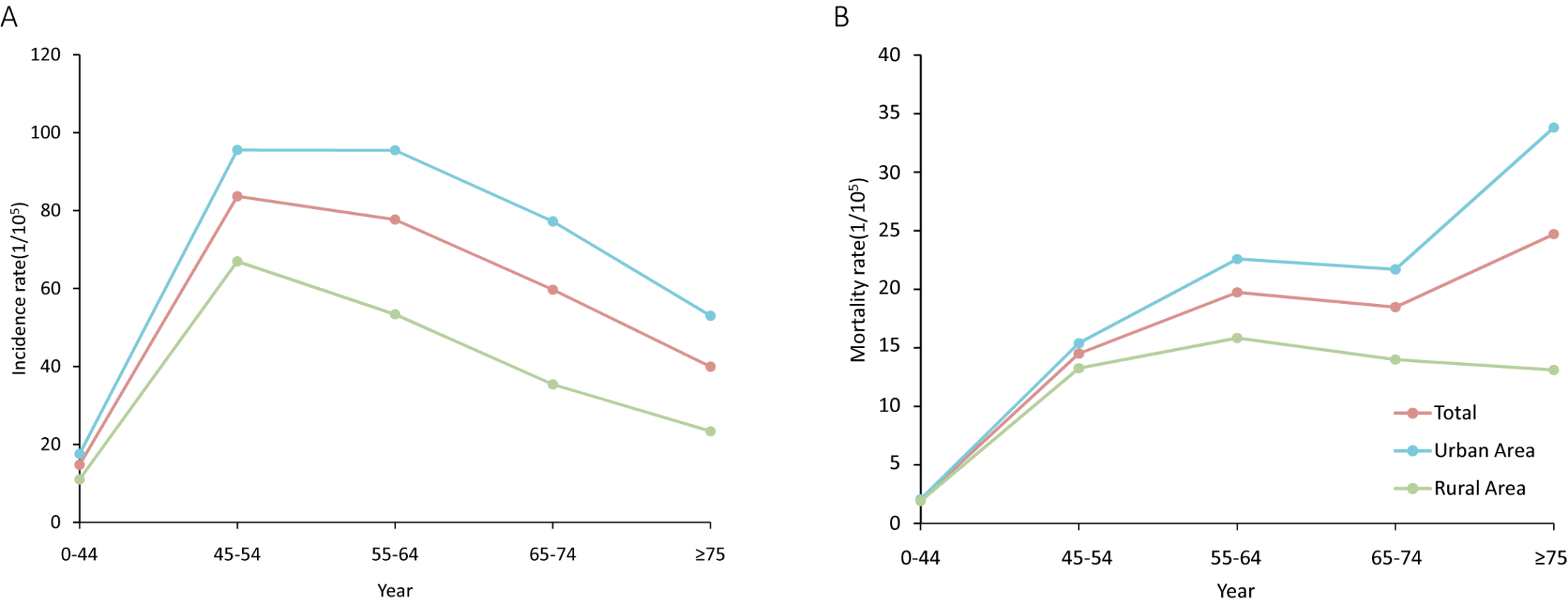
**

**Figure S1** Incidence and mortality rate of female breast cancer in 2011-2020, by sex. (A) Incidence; (B) Mortality.
